# Diffuse hair loss in a patient with systemic lupus erythematosus

**DOI:** 10.1016/j.jdcr.2023.07.032

**Published:** 2023-08-08

**Authors:** Sahana Ummadi, Shelley Stepenaskie, Sharon E. Nunez, Nikifor K. Konstantinov

**Affiliations:** a University of New Mexico School of Medicine, Albuquerque, New Mexico; b Department of Dermatology, University of New Mexico School of Medicine, Albuquerque, New Mexico; c Rheumatology Division, Department of Internal Medicine, University of New Mexico School of Medicine, Albuquerque, New Mexico

**Keywords:** alopecia, fibrosing alopecia in a pattern distribution, frontal fibrosing alopecia, lupus, lupus hair loss

## History

**Fig 1:**
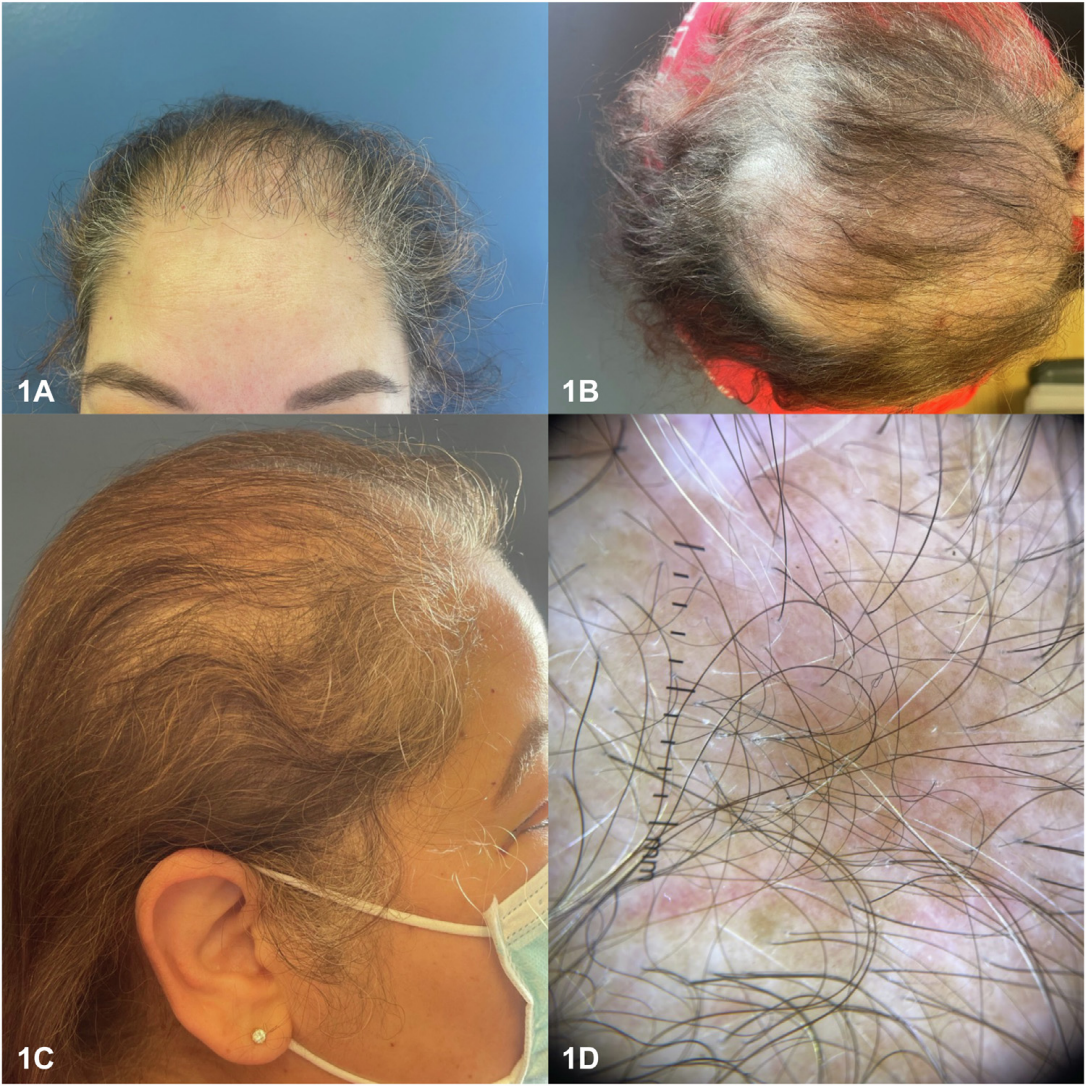

A 57-year-old woman with a history of systemic lupus erythematosus presented to a dermatology clinic with 20 years of ongoing hair loss affecting the frontoparietal scalp and crown (Fig 1, *A-D*). The patient denied scalp itch, pain, and was otherwise asymptomatic.

**Fig 2:**
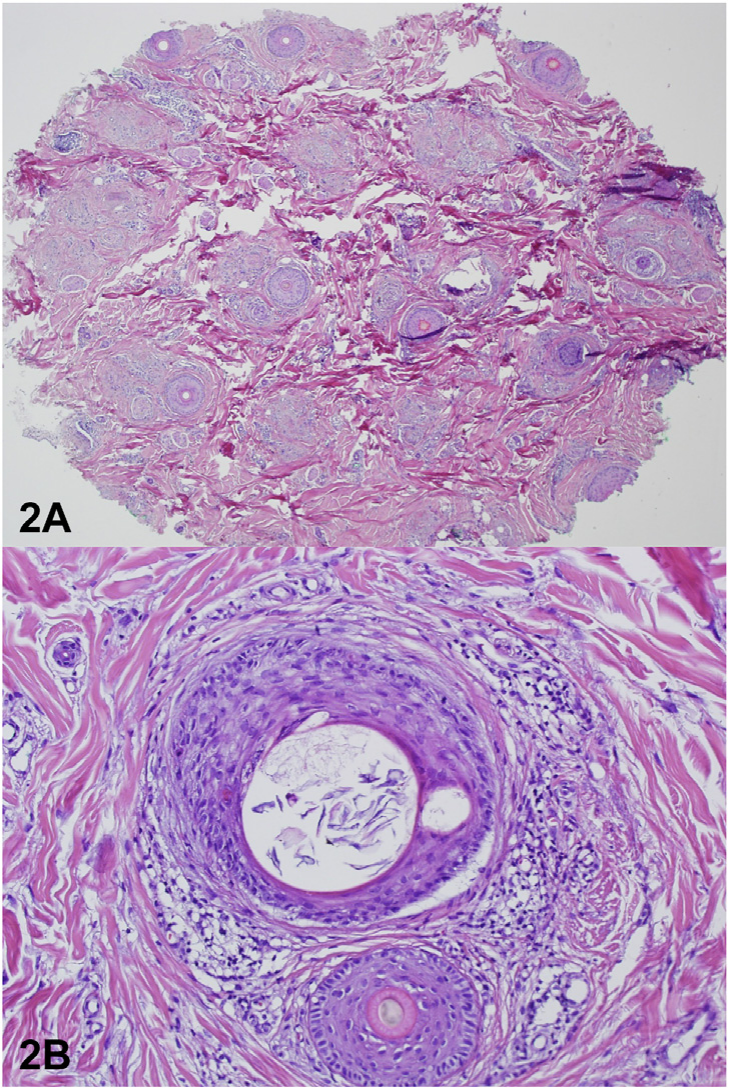

A punch biopsy was performed of the vertex scalp which showed decrease and miniaturization in the number of hair follicles, associated with sebaceous glands, fibrous stellae, and a peri-infundibular lymphocytic infiltrate with few dyskeratotic keratinocytes (Fig 2, *A* and *B*).

The patient was started on hydroxychloroquine, methotrexate, topical clobetasol, oral minoxidil, and intralesional Kenalog injections at her subsequent visits, with improvement in hair regrowth.

**Question 1: What is the most likely diagnosis?**

A. Frontal fibrosing alopecia (FFA)
B. Alopecia Areata
C. Lupus hair
D. Fibrosing alopecia with a pattern distribution (FAPD)
E. Discoid lupus erythematosus (DLE)

**Answers:**

A. FFA – Incorrect. The clinical presentation does not support this diagnosis. Frontal fibrosing alopecia (FFA) is a lymphocytic cicatricial alopecia characterized by frontotemporal and eyebrow hairloss.^1^ FFA is also associated with facial papules. FFA has been reported to occur rarely in both discoid lupus and systemic lupus erythematosus.^1^
B. Alopecia Areata – Incorrect. Diffuse alopecia areata may be a mimicker clinically, however the classic histopathology would not show a scarring alopecia. There would be absence of perifollicular fibrosis and there would be a lymphocytic cell infiltrate around the anagen bulb.^2^
C. Lupus hair – Incorrect. Lupus hair is characterized by dry and fragile short hairs on the frontal hair-line in patients with SLE, giving the appearance of vellus hairs on the anterior scalp.^2^
D. FAPD – Correct. FAPD is a lymphocytic cicatricial alopecia that has been recently described and has overlapping clinical and histopathologic features of FFA and androgenetic alopecia.^3^ Our patient has hair loss in a frontoparietal distribution with histopathology showing lymphocytic infiltrate around the infundibular region of the hair follicle, perifollicular lamellar fibrosis, and miniaturized hair follicles which makes FAPD most likely.^3^ FAPD occurs more frequently in postmenopausal women than men. White patients are more commonly affected, but FAPD has been reported in Hispanic individuals and those of African descent.^3^
E. DLE – Incorrect. Discoid lupus can cause a scarring alopecia characterized by violaceous atrophic plaques with follicular plugging and dyspigmentation. Histopathology shows interface dermatitis, basement membrane thickening, lymphohistiocytic infiltration around vessels and appendages, mucin deposition, and follicular keratotic plugs.^2^

**Question 2: What are the classic trichoscopic findings in FAPD?**

A. Loss of follicular openings, hair diameter variability, and perifollicular hyperkeratosis
B. Yellow dots and tapered hairs
C. Loss of hair follicles, yellow dots, and dystrophic hairs
D. Black dots, comma hairs, and scale
E. Arborising telangiectasia, dark brown pigmentation, and follicular plugs

**Answers:**

A. Loss of follicular openings, hair diameter variability, and perifollicular hyperkeratosis – Correct. The classic trichoscopic findings of FAPD include perifollicular hyperkeratosis, loss of follicular ostia, and hair diameter variability (Fig 1, *D*). Other findings may include perifollicular erythema, hair tufting, and predominance of single hair follicles.^3^
B. Yellow dots and tapered hairs – Incorrect. Yellow dots and tapered hairs are indicative of alopecia areata, which can also present with dystrophic and fractured telogen roots.^4^
C. Loss of hair follicles, yellow dots, and dystrophic hairs – Incorrect. Loss of hair follicles, yellow dots and dystrophic hairs are indicative of dissecting cellulitis, which can also present with follicular pustules at the borders.^4^
D. Black dots, comma hairs, and scale – Incorrect. Black dots, comma hairs, and scale are indicative of tinea capitis, which can also present with blotchy pigmentation.^4^
E. Arborising telangiectasia, dark brown pigmentation, and follicular plugs – Incorrect. Arborising telangiectasia, dark brown pigmentation, and follicular plugs are indicative of DLE.^4^

**Question 3: Which of the following is not a recommended therapy for a patient with advanced FAPD at this time?**

A. Topical corticosteroids
B. Topical minoxidil
C. Hydroxychloroquine
D. Hair transplantation
E. Antiandrogen therapy

**Answers:**

A. Topical corticosteroids – Incorrect. Topical corticosteroids are appropriate to use, however when used alone do not appear to affect hair loss but may improve symptoms of FAPD.^3^ Report of improvement has been seen with topical clobetasol proprionate solution and triamcinolone acetonide 0.2% solution, in combination with topical 5% minoxidil and hydroxychloroquine.^3^
B. Topical minoxidil – Incorrect. Topical minoxidil is a common treatment used in androgenetic alopecia and also to help thicken miniaturized hairs.^3^ Topical minoxidil has been used in combination with other therapies for treatment of FAPD.^3^
C. Hydroxychloroquine – Incorrect. Hydroxychloroquine is commonly used in lichen planopilaris (LPP) and FFA. It has been reported to be successful in a cohort of 4 patients with FAPD in combination with topical clobetasol proprionate and topical 5% minoxidil.^3^ Though there are limited data regarding its use in FAPD, the patient has SLE, so it is an appropriate medication to use.
D. Hair transplantation – Correct. There is no good evidence to support successful treatment of FAPD with hair transplantation. LPP and FFA have been seen following this procedure in a cohort of 10 patients.^5^ Given the clinical and histopathologic similarities between these conditions, hair transplant would not be recommended at this time.
E. Antiandrogen therapy – Incorrect. Antiandrogen therapy (cyproterone and finasteride) have been evaluated in case series used in combination with topical corticosteroids and topical 5% minoxidil to improvement hair regrowth in FAPD.^3^ Although our patient is not on antiandrogen therapy, it would be a reasonable treatment to add should her hair loss progress on current therapy.

## Conflicts of interest

None disclosed.
